# TALE‐carrying bacterial pathogens trap host nuclear import receptors for facilitation of infection of rice

**DOI:** 10.1111/mpp.12772

**Published:** 2019-01-09

**Authors:** Shugang Hui, Yarui Shi, Jingjing Tian, Li Wang, Yueyue Li, Shiping Wang, Meng Yuan

**Affiliations:** ^1^ National Key Laboratory of Crop Genetic Improvement National Center of Plant Gene Research (Wuhan), Huazhong Agricultural University Wuhan 430070 China

**Keywords:** nuclear import receptor, nuclear localization signals, rice, transcription activator‐like effector, *Xanthomonas*

## Abstract

Many plant‐pathogenic *Xanthomonas* rely on the secretion of virulence transcription activator‐like effector (TALE) proteins into plant cells to activate plant susceptibility genes to cause disease. The process is dependent on the binding of TALEs to specific elements of host target gene promoters in the plant nucleus. However, it is unclear how TALEs, after injection into host cells, are transferred from the plant cytoplasm into the plant nucleus, which is the key step of successful pathogen infection. Here, we show that the host plant cytoplasm/nuclear shuttle proteins OsImpα1a and OsImpα1b are key components for infection by the TALE‐carrying bacterial pathogens *Xanthomonas oryzae* pv. *oryzae* (*Xoo*) and *Xanthomonas oryzae* pv. *oryzicola *(*Xoc*), the causal agents of bacterial leaf blight and bacterial leaf streak, respectively, in rice. Direct interaction between the second nuclear localization signal of TALEs of *Xoo* or *Xoc* and OsImpα1a or OsImpα1b is required for the transportation of TALEs into the nucleus. Conversely, suppression of the expression of *OsImp*α*1a *and *OsImp*α*1b* genes attenuates the shuttling of TALEs from the cytoplasm into the nucleus and the induction of susceptibility genes, thus improving the broad‐spectrum disease resistance of rice to *Xoo* and *Xoc*. These results provide an applicable strategy for the improvement of resistance to TALE‐carrying pathogens in rice by moderate suppression of the expression of plant nuclear import receptor proteins.

## Introduction

*Xanthomonas*, which is a large group of Gram‐negative bacterial plant pathogens, consists of almost 30 species and causes diseases on at least 124 monocotyledonous and 268 dicotyledonous plants (Ryan *et al.*, 2011). Many *Xanthomonas* bacteria secrete transcription activator‐like effectors (TALEs), the major virulence and determinant factors in host–pathogen interactions, into plant cells, where they act as transcriptional activators to reprogram the host plant transcriptome. TALEs function as eukaryotic transcription factors, which activate the transcription of host susceptibility genes to cause susceptibility of plants or induce the expression of host resistance genes to cause resistance of plants, and rely on their injection into the plant nucleus and their binding to the effector binding element (EBE) of host target gene promoters (Cox *et al.*, 2017; Zhang *et al.*, 2015). TALEs are highly conserved, with homologues across *Xanthomonas* species sharing greater than 90% amino acid identity and almost identical structural features. In general, TALEs typically consist of the following: an N‐terminal type III secretion signal that guides the translocation of TALEs from the bacterium into the host plant cytoplasm through the type III secretion system (T3SS); a central repeat region (RR), which is the main hallmark of TALEs, that specifically binds to EBE of the host gene promoter; a transcription factor binding (TFB) motif that hijacks the host plant basal transcription factor IIA γ subunit to fulfil the binding of TALEs to EBE; three short nuclear localization signals (NLSs) that guide the translocation of TALEs from the plant cytoplasm into the plant nucleus; and a highly conserved acidic activation domain (AD) that allows TALEs to activate gene transcription in plant cells (Doyle *et al.*, 2013; Huang *et al.*, 2017; Yuan *et al.*, 2016; Zhang *et al.*, 2015).

*Xanthomonas oryzae* pv. *oryzae* (*Xoo*) causes bacterial leaf blight of rice and *Xanthomonas oryzae* pv. *oryzicola *(*Xoc*) causes bacterial leaf streak of rice, both of which are devastating bacterial diseases. Massive efforts have been undertaken to decipher *Xoo *and *Xoc* TALE biology. Numerous susceptibility genes in rice targeted by TALEs have been forecasted and validated. TALEs pthXo1, pthXo2 and pthXo3/AvrXa7/Tal5/TalC/TalF of *Xoo* induce the expression of *Xa13*/*Os8N3*/*SWEET11*, *Xa25*/*Os12N3*/*SWEET13* and *Xa41*/*Os11N3*/*SWEET14*, respectively, which are three members of the rice MtN3/saliva/SWEET family and function as sucrose transporters (Antony *et al.*, 2010; Blanvillain‐Baufumé *et al.*, 2017; Chu *et al.*, 2006; Hutin *et al.*, 2015; Liu *et al.*, 2011; Streubel *et al.*, 2013; Tran *et al.*, 2018; Yang *et al.*, 2006; Zhou *et al.*, 2015). TALEs pthXo6, TalB and pthXo7 of *Xoo* up‐regulate the transcription factors *OsTFX1*, *OsERF#123* and *OsTFIIAγ1*, respectively (Sugio *et al.*, 2007; Tran *et al.*, 2018). TALEs Tal9a of *Xoo* and Tal1c of *Xoc *trigger the induction of *OsHen1*, which encodes a protein with a predicted methyltransferase domain involved in micro‐RNA (miRNA) maturation (Moscou and Bogdanove, 2009). TALE Tal2g of *Xoc *activates *OsSULTR3;6*, which encodes a sulfate transporter (Cernadas *et al.*, 2014). All of these susceptibility genes can benefit *Xoo* or *Xoc* multiplication and disease development after activation by TALEs, although they encode different types of protein. *Xoo* and *Xoc* contain 8–26 and 19–29 TALEs, respectively, based on genome sequence (Booher *et al.*, 2015; Cernadas *et al.*, 2014). In theory, each TALE specifically binds to the sole EBE of the host target gene promoter to activate its transcription. During this process, the *Xoo*‐ and *Xoc*‐derived TALEs, after injection into plant cells, must be actively transported into the plant nucleus to bind to the target susceptibility gene promoter to reprogram the transcriptome of rice to cause disease. However, the underlying mechanism of how TALEs are transferred from the plant cytoplasm into the plant nucleus is still unclear.

So far, multiple pathways of nucleocytoplasmic transport have been identified, each likely to be involved in carrying a specific group of proteins (Goldfarb *et al.*, 2004; Peters, 2006), the best characterized of which is the import of proteins containing a classical NLS that consists of either a short stretch of three to five basic amino acids or two basic domains separated by a spacer. The NLS‐containing proteins are recognized and bound in the cytoplasm by the NLS receptor, and then transported into the nucleus. In rice, importin proteins OsImpα1a and OsImpα1b, which function as nucleocytoplasmic transporters, selectively bind and transport proteins containing T‐NLS (a monopartite‐type NLS with typical amino acid residues CTPPKKKRKV) and O_2_‐NLS (a bipartite‐type NLS with typical amino acid residues MPTEERVRKRKESNRESARRSRYRKAAHLKC), but not R‐NLS (a yeast Matα‐2‐type NLS with typical amino acid residues CYMISEALRKAIGKR) (Chang *et al.*, 2012, 2014; Goldfarb *et al.*, 2004; Jiang *et al.*, 1998, 2001). Here, we reveal that rice OsImpα1a and OsImpα1b coordinately transfer the TALEs of *Xoo* and *Xoc* from the plant cytoplasm into the nucleus by selectively binding the bacterial pathogen‐derived NLS of TALEs, which features a short stretch of five amino acids rich in arginine and lysine residues (RKRSR). We further demonstrate that OsImpα1a and OsImpα1b (cytoplasm/nucleus shuttle proteins)‐mediated transportation of TALEs is vital for *Xoo‐* and *Xoc*‐triggered induction of targeting of susceptibility genes. Our results suggest that modification of the host nuclear import receptor genes *OsImp*α*1a* and *OsImp*α*1b* of rice by moderate suppression may provide a universally applicable strategy to improve plant resistance to the TALE‐carrying bacterial pathogens *Xoo* and *Xoc*.

## Results

### Rice OsImpα1a interacts with *Xoo *pthXo1

Like other *Xoo* TALEs, pthXo1 also typically contains an amino‐terminal translocation signal (TS), a central RR, a newly identified TFB motif, three NLSs and a carboxyl‐terminal transcription AD. Our previous studies have demonstrated that the complete pthXo1 possesses auto‐activation activity because of the presence of the TS or AD domain; the truncated pthXo1 containing RR‐TFB‐NLS does not show auto‐activation transcription activity in yeast (Yuan *et al.*, 2016). To screen for pthXo1‐interacting host rice proteins, the RR‐TFB‐NLS fragment of pthXo1 was used as a bait to trap proteins putatively interacting with pthXo1 by yeast two‐hybrid assay. The prey cDNA library was prepared from mRNA isolated from leaves of rice variety IR24 after inoculation with *Xoo* strain PXO99. Several candidate pthXo1‐interacting proteins were identified from approximately 5 × 10^5^ independent colonies screened. Among the putative interacting proteins, the cDNA for the rice importin α1a gene (hereafter designated as *OsImpα1a*) showed the strongest interaction with the RR‐TFB‐NLS fragment of pthXo1. Rice *OsImpα1a* encodes a protein of 526 amino acids and has high amino acid sequence similarity with its homologues in rice, such as OsImpα1b and OsImpα2 (Jiang *et al.*, 1998, 2001). A phylogenetic tree of plant importin α proteins, obtained from *Xanthomonas*‐invaded host plants, showed that OsImpα1a grouped with Arabidopsis AtImpα1 and AtImpα2, pepper CaImpα2 and citrus CsImpα2 (Fig. S1, see Supporting Information); CaImpα2 has been shown to interact with *Xanthomonas campestris* pv. *vesicatoria* (*Xcv*) TALE AvrBs3 in yeast (Szurek *et al.*, 2001).

### pthXo1 interacts with OsImpα1a and OsImpα1b through NLS2

**Figure 1:**
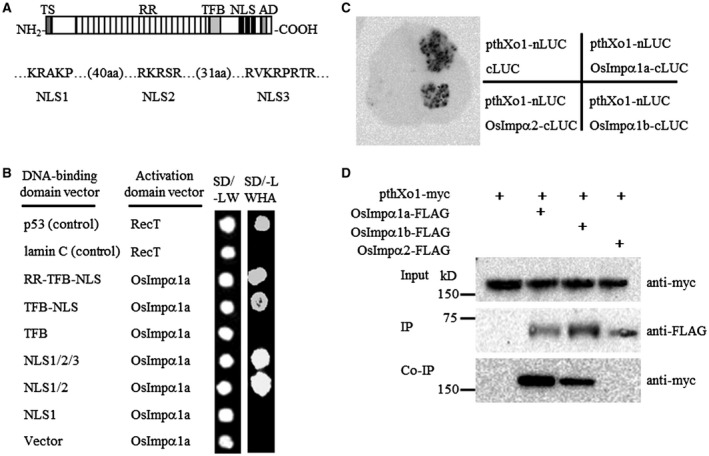
*Xanthomonas oryzae* pv. *oryzae* (*Xoo*) pthXo1 interacts with rice OsImpα1a and OsImpα1b. (A) The conserved structures of pthXo1. TS, amino‐terminal translocation signal; RR, central repeat region; TFB, transcription factor binding region; NLS, nuclear localization signal; AD, carboxyl‐terminal transcription activation domain. (B) The NLS2 of pthXo1 is required for the interaction with OsImpα1a analysed by yeast two‐hybrid assay. The interactions were assessed by the growth of yeast cells on synthetic defined premixed (SD) medium lacking (–) leucine (L), tryptophan (W), histidine (H) and adenine (A). Vector, empty vector as control. (C) Firefly luciferase (LUC) complementation imaging assay. *Nicotiana benthamiana *leaves were co‐infiltrated with agrobacterial strains containing different pairs of constructs. LUC images were captured using a cooled charge coupled device (CCD) imaging apparatus. The grey shape is one *N. benthamiana *leaf. The black circle indicates an interaction between two proteins. (D) Detection of interactions between *Xoo* pthXo1 and rice OsImpα1a or OsImpα1b *in planta *by co‐immunoprecipitation (Co‐IP). The protein–protein interaction assays were performed in *N. benthamiana *leaf cells. Proteins before (Input) and after immunoprecipitation (IP) were detected with anti‐myc and anti‐FLAG antibodies.

To narrow down which domain of pthXo1 interacts with OsImpα1a, we generated a series of pthXo1 constructs in which truncated coding sequences of *pthXo1* were translationally fused with the DNA‐binding domain of GAL4. These constructs included the entire RR to NLSs (RR‐TFB‐NLS), the TFB motif with NLSs (TFB‐NLS), TFB, the three NLSs (NLS1/2/3), the first and second NLSs (NLS1/2) and the three different NLSs (NLS1, NLS2, NLS3) (Fig. 1A). The resulting constructs were co‐transformed into yeast cells with the complete coding sequence of OsImpα1a fused with the DNA AD of GAL4, and empty vectors as the negative controls. The second NLS (NLS2) was the only domain to interact with OsImpα1a in the yeast two‐hybrid assay (Fig. 1B). In contrast, RR, TFB, NLS1 and NLS3 did not exhibit interaction with OsImpα1a in yeast (Fig. S2A, see Supporting Information). Further, we found that NLS2 of pthXo1 also interacted with OsImpα1b, but not with OsImpα2, in yeast two‐hybrid assay (Fig. S2B,C). These results demonstrate that the second NLS of pthXo1 is necessary for the interaction with the two rice nuclear import receptor proteins OsImpα1a and OsImpα1b.

### pthXo1 interacts with OsImpα1a and OsImpα1b *in vivo*

To validate the yeast two‐hybrid results, the interaction between pthXo1 and OsImpα1a or OsImpα1b was verified by *in vivo* assay. We first performed a firefly split‐luciferase complementation assay in *Nicotiana benthamiana* leaves to investigate the interaction. During this assay, pthXo1 was fused with the N‐terminus (nLUC) of firefly luciferase; OsImpα1a, OsImpα1b and OsImpα2 were fused with the C‐terminus (cLUC) of firefly luciferase. We transiently co‐expressed pthXo1‐nLUC and OsImpα1a‐cLUC, pthXo1‐nLUC and OsImpα1b‐cLUC, pthXo1‐nLUC and OsImpα2‐cLUC, as well as pthXo1‐nLUC and cLUC as negative controls, in *N. benthamiana* leaves. A strong fluorescence signal was observed in leaves that co‐expressed pthXo1‐nLUC and OsImpα1a‐cLUC, or pthXo1‐nLUC and OsImpα1b‐cLUC, but not in pthXo1‐nLUC and OsImpα2‐cLUC, or negative controls (Fig. 1C), suggesting that pthXo1 interacted with OsImpα1a and OsImpα1b, but not OsImpα2, *in planta*.

We then performed co‐immunoprecipitation (Co‐IP) assays using pthXo1‐myc and OsImpα1a‐FLAG, OsImpα1b‐FLAG or OsImpα2‐FLAG co‐expressed transiently in *N. benthamiana* leaves. The OsImpα1a‐FLAG, OsImpα1b‐FLAG and OsImpα2‐FLAG proteins were immunoprecipitated using anti‐FLAG‐conjugated agarose. Immunoblots were washed and probed with anti‐myc antibodies. The pthXo1‐myc protein was pulled down by OsImpα1a‐FLAG and OsImpα1b‐FLAG. In contrast, OsImpα2‐FLAG could not pull down pthXo1‐myc protein in the same conditions (Fig. 1D). Taken together, these results demonstrate that *Xoo* TALE pthXo1, via the second NLS, physically associates with host rice OsImpα1a and OsImpα1b.

### NLS2 of TALE is essential for *Xoo* virulence

**Figure 2:**
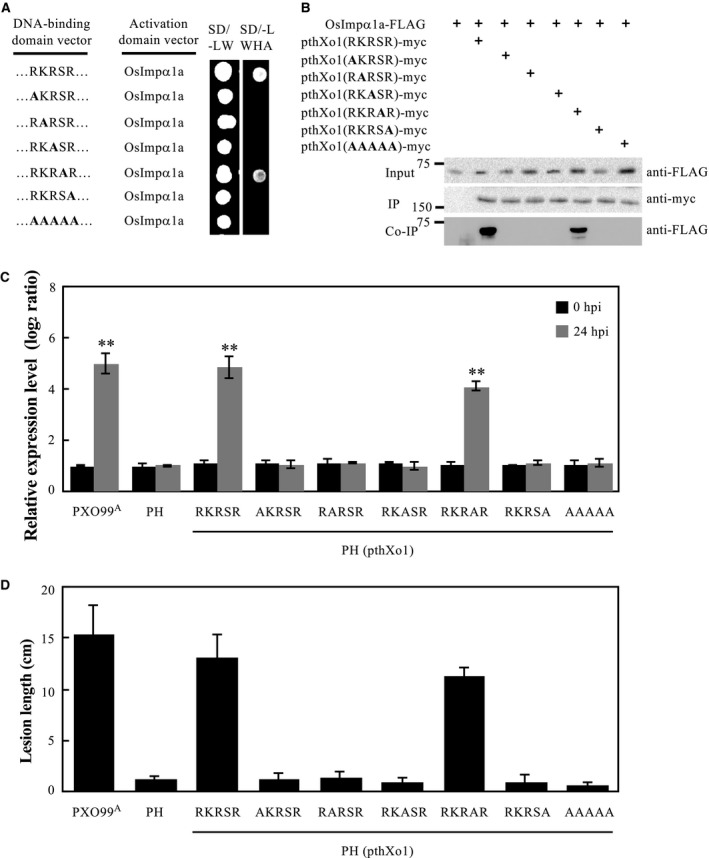
Effect of transcription activator‐like effector (TALE) pthXo1 NLS2 on the virulence of *Xanthomonas oryzae* pv. *oryzae* (*Xoo*) strains. (A) Interaction between point‐mutated NLS2 of pthXo1 and OsImpα1a analysed by yeast two‐hybrid assay. The interactions were assessed by the growth of yeast cells on synthetic defined premixed (SD) medium lacking (–) leucine (L), tryptophan (W), histidine (H) and adenine (A). (B) Detection of interactions between different mutated pthXo1 and OsImpα1a *in planta *by co‐immunoprecipitation (Co‐IP). The protein–protein interaction assays were performed in *Nicotiana benthamiana *leaf cells. Protein before (Input) and after immunoprecipitation (IP) were detected with anti‐myc and anti‐FLAG antibodies. (C) Expression of pthXo1 targeting susceptibility gene *Xa13* after infection of different strains. Asterisks indicate a significant difference between non‐infected plants and *Xoo*‐infected plants at **P < 0.01. hpi, hours post‐inoculation. (D) Virulence of strain PH and its derivatives carrying pthXo1 or NLS2‐mutated pthXo1 in rice. PH is an engineered TALE‐free strain with the genetic background of strain PXO99^A^, which carries pthXo1.

We confirmed that *Xoo* TALE pthXo1 interacts with OsImpα1a and OsImpα1b *in vitro* and *in vivo*. We narrowed down the core interaction region of pthXo1 to NLS2. When aligning the NLS2 of TALEs from genome sequenced *Xoo* and *Xoc *strains, we found that all of the TALEs of different *Xoo* and *Xoc* strains exclusively have the same amino acid residues of their NLS2 (RKRSR). To assess which amino acid residues of RKRSR in NLS2 are essential for TALE function or *Xoo* virulence, we first produced pthXo1 derivatives with the five conserved amino acid residues of NLS2 substituted by alanine, individually. Of the NLS2 forms of pthXo1 tested, those with RKRSR and RKRAR interacted with OsImpα1a, but those with AKRSR, RARSR, RKASR and RKRSA did not, in yeast two‐hybrid assay (Fig. 2A). The same interaction was also observed between OsImpα1b and pthXo1 with NLS2 containing RKRSR or RKRAR, but not with the other mutations (Fig. S3, see Supporting Information). We then validated the interaction *in vivo*. An *in* *planta* Co‐IP assay strongly demonstrated that the substitution of the NLS2 residues of pthXo1 completely destroyed its interaction with OsImpα1a (Fig. 2B).

To learn whether the conserved amino acid residues in NLS2 of TALE are directly responsible for the expression of TALE‐induced host plant susceptibility genes and virulence on host rice, we generated NLS2 substituted with alanine in pthXo1. The constructs were re‐introduced into *Xoo* strain PH, which is an engineered TALE‐free strain with the genetic background of PXO99^A^ (Ji *et al.*, 2016). Simultaneously, pthXo1(RKRSR) was introduced into PH as a control. After inoculation of rice variety IR24 with these *Xoo* strains, the expression of *Xa13*, the corresponding target gene of pthXo1, was activated by the presence of pthXo1 with RKRSR or RKRAR in NLS2, but not by versions of pthXo1 with AKRSR, RARSR, RKASR or RKRSA in NLS2 (Fig. 2C). The lesion length in these experiments was correlated with the up‐regulated expression level of susceptibility gene *Xa13* (Fig. 2D). In summary, these data show that the conserved amino acid residues of NLS2 of TALEs play key roles in the transcription of TALE‐induced host plant susceptibility genes and *Xoo* virulence on host rice.

### Localization of OsImpα1a and OsImpα1b

**Figure 3:**
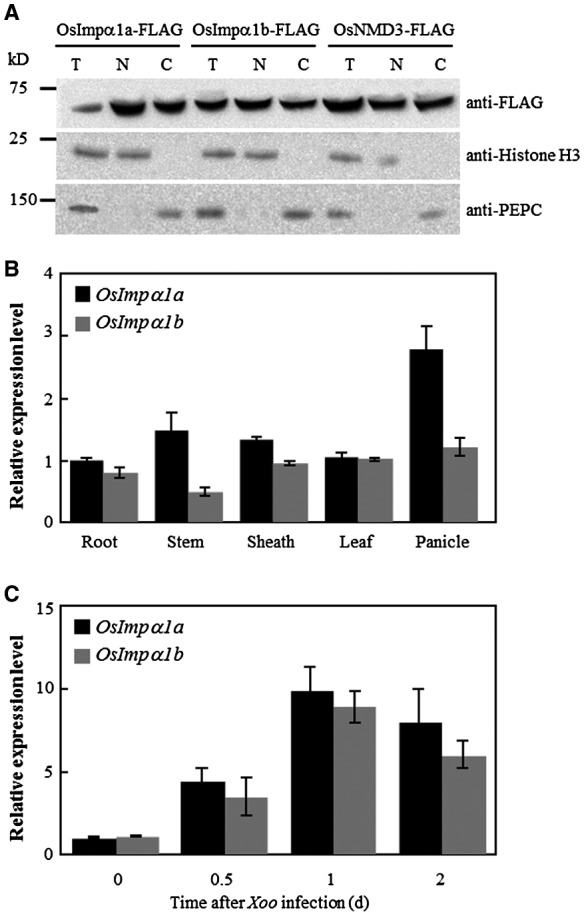
Expression patterns of *OsImpα1a* and *OsImpα1b*. (A) OsImpα1a and OsImpα1b are nucleocytoplasmic‐localized proteins. Total protein, the nucleus‐depleted fraction and nucleus‐enriched fraction were loaded onto a sodium dodecylsulfate‐polyacrylamide gel electrophoresis (SDS‐PAGE) gel and subjected to immunoblot analysis. Histone H3 and phosphoenolpyruvate carboxylase (PEPC) were used as nuclear and cytosolic markers, respectively. T, total protein extracts; N, nucleus‐enriched fraction; C, nucleus‐depleted fraction. (B) Expression of *OsImpα1a* and *OsImpα1b *in different tissues. Tissues were collected at the booting stage from IR24. (C) Expression of *OsImpα1a* and *OsImpα1b* after infection with *Xanthomonas oryzae* pv. *oryzae* (*Xoo*) strain PXO99.

To investigate the intracellular location of OsImpα1a and OsImpα1b, we transiently expressed OsImpα1a‐FLAG and OsImpα1b‐FLAG fusion constructs, as well as OsNMD3‐FLAG as a nucleocytoplasmic‐localized protein control (Shi *et al.*, 2014), into *N. benthamiana* leaves. We performed subcellular fractionation analyses, followed by immunoblotting using FLAG antibody, accompanied by histone H3 antibody and phosphoenolpyruvate carboxylase (PEPC) antibody, which have been used as nuclear and cytosolic markers, respectively. The OsImpα1a‐FLAG and OsImpα1b‐FLAG proteins were detected in total protein extracts, the nucleus‐enriched fraction and the nucleus‐depleted fraction, in accordance with the nucleus and cytoplasm shuttle protein OsNMD3 (Fig. 3A). Taken together, these data indicate that OsImpα1a and OsImpα1b are located in the nucleus and cytoplasm, suggesting their roles as cytoplasm/nuclear shuttle proteins to transfer proteins between the cytoplasm and nucleus.

### Expression patterns of *OsImpα1a *and *OsImpα1b*

To determine the expression patterns of *OsImpα1a* and *OsImpα1b*, we sampled the five tissues (root, stem, sheath, leaf and panicle) of IR24 at the booting stage, and assessed the transcript levels of the two genes in these tissues. Quantitative reverse transcription‐polymerase chain reaction (qRT‐PCR) analyses showed that *OsImpα1a* and *OsImpα1b* were constitutively expressed in all of these tissues, with *OsImpα1a* and *OsImpα1b* having relatively higher transcription levels in the panicle compared with other tissues. *OsImpα1a* showed slightly higher expression than *OsImpα1b *in the different tissues (Fig. 3B).

We then tested the transcript accumulation of *OsImpα1a* and *OsImpα1b* in response to bacterial pathogen infection. *OsImpα1a* and *OsImpα1b* accumulation was rapidly and markedly induced after *Xoo* infection in the leaf tissue of IR24, which is susceptible to *Xoo* strain PXO99 (Fig. 3C). In addition, *OsImpα1a* and *OsImpα1b* transcription was significantly activated after *Xoc* infection (Fig. S4, see Supporting Information), suggesting that *OsImpα1a* and *OsImpα1b* transcription occurs in response to bacterial pathogen infection.

### *OsImpα1a *and *OsImpα1b* influence resistance to bacterial pathogens

**Figure 4:**
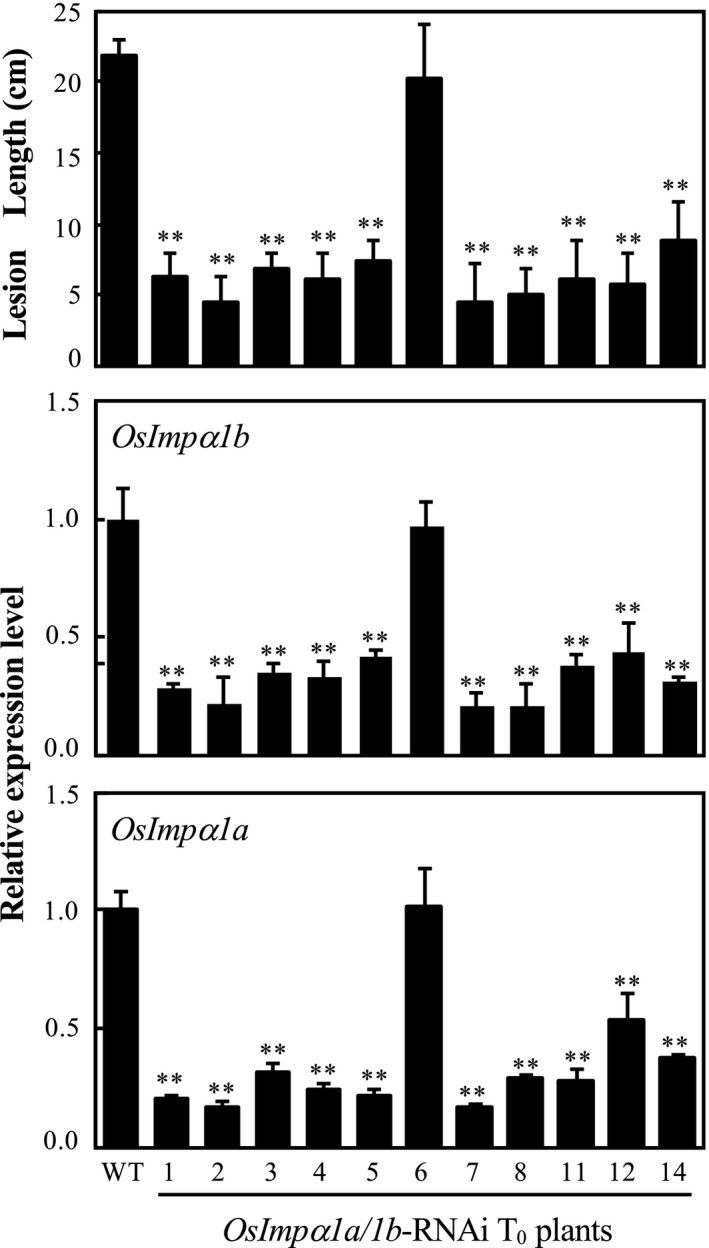
Modulation of *OsImpα1a* and *OsImpα1b *expression influences rice response to *Xanthomonas oryzae* pv. *oryzae* (*Xoo*) infection. Analysis of the response of *OsImpα1a/1b*‐RNAi T_0_ plants to *Xoo* strain PXO99. Data represent the mean (five to eight leaves from one plant for lesion length) ± standard deviation (SD). Asterisks indicate a significant difference between transgenic plants and wild‐type (WT) IR24 at **P < 0.01.

**Figure 5:**
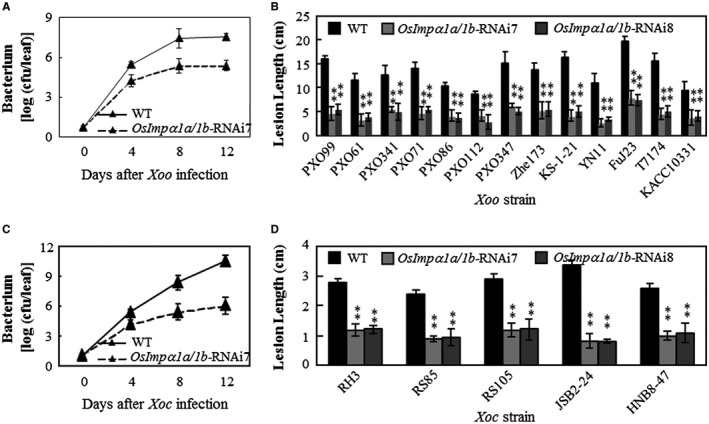
Effect of *OsImpα1a *and *OsImpα1b* on rice–bacterial pathogen interaction. Plants were inoculated with *Xanthomonas oryzae* pv. *oryzae* (*Xoo*) at the booting stage and *Xanthomonas oryzae* pv. *oryzicola *(*Xoc*) at the tillering stage. (A) Growth of *Xoo* strain PXO99 in leaves of *OsImpα1a/1b*‐RNAi plants (T_2_ generation). Data represent the mean (nine leaves from three plants) ± standard deviation (SD). cfu, colony‐forming unit. (B) The responses of *OsImpα1a/1b*‐RNAi plants (T_3_ generation) to different *Xoo* strains. Data represent the mean (12–15 leaves from three plants) ± SD. (C) Growth of *Xoc* strain RS105 in leaves of *OsImpα1a/1b*‐RNAi plants (T_2_ generation). Data represent the mean (nine leaves from three plants) ± SD. (D) The responses of *OsImpα1a/1b*‐RNAi plants (T_3_ generation) to different *Xoc* strains. Data represent the mean (12–15 leaves from three plants) ± SD. WT, wild‐type.

To determine whether *OsImpα1a* and *OsImpα1b* play a role in rice–bacterial pathogen interaction, *OsImpα1a‐* and *OsImpα1b*‐suppressing plants were generated using an RNA interference (RNAi) strategy. Because *OsImpα1a* and *OsImpα1b* showed similar expression patterns in diverse tissues and after bacterial pathogen infection, and had high nucleotide identity at 77% at the mRNA level (Fig. S5, see Supporting Information), it was difficult to choose a gene‐specific fragment for the RNAi construct to target individual genes; therefore, we chose the common region of *OsImpα1a* and *OsImpα1b* to generate the RNAi construct to simultaneously suppress these two homologous genes. After the *OsImpα1a/1b*‐RNAi construct had been transformed into IR24, which is susceptible to a large number of *Xoo* and *Xoc* strains, 11 independent transgenic plants were obtained. The expression of *OsImpα1a* and *OsImpα1b* was significantly reduced in 10 of the 11 plants. All of the transgenic plants were inoculated with *Xoo* strain PXO99 at the booting stage. The transgenic plants with lower *OsImpα1a* and *OsImpα1b* expression levels showed remarkably enhanced resistance to PXO99 with lesion lengths of 4.5 ± 2.5 cm to 8.8 ± 2.7 cm, compared with the wild‐type lesion length of 21.8 ± 1.1 cm (Fig. 4). Increased resistance to *Xoo* was associated with reduced *OsImpα1a* and *OsImpα1b* expression, which was further confirmed in two T_1_ families. T_1_ families from two independent *OsImpα1a/1b*‐RNAi T_0_ plants with reduced expression of *OsImpα1a* and *OsImpα1b* were inoculated with *Xoo* at the booting stage. Some of the transgenic plants showed significantly enhanced resistance compared with the wild‐type, and this resistance was associated with suppressed expression of *OsImpα1a* and *OsImpα1b* (Fig. S6, see Supporting Information). The reduction in *Xoo*‐related disease symptoms in *OsImpα1a/1b*‐RNAi plants was significantly correlated with the reduced expression of *OsImpα1a* and *OsImpα1b* in the *OsImpα1a/1b*‐RNAi7 T_1_ family (*r* = 0.989 for *OsImpα1a*, *r* = 0.992 for *OsImpα1b*, *n* = 8, *α* < 0.01) and the *OsImpα1a/1b*‐RNAi8 T_1_ family (*r* = 0.981 for *OsImpα1a*, *r* = 0.968 for *OsImpα1b*, *n* = 10, *α* < 0.01). Moreover, the *Xoo* growth rates in the leaves of *OsImpα1a/1b*‐RNAi plants were observably lower than those in the wild‐type (Fig. 5A). Furthermore, to assess whether *OsImpα1a/1b*‐RNAi plants enhance resistance to other *Xoo* strains, we inoculated *OsImpα1a/1b*‐RNAi7 and *OsImpα1a/1b*‐RNAi8 T_2_ families with 13 *Xoo* strains containing seven Philippine strains, four Chinese strains, one Japanese strain and one Korean strain, which are commonly used to test for broad‐spectrum resistance to bacterial leaf blight. We found that *OsImpα1a/1b*‐RNAi plants showed broad‐spectrum resistance to the different *Xoo* strains (Fig. 5B).

In addition, we examined the resistance of *OsImpα1a/1b*‐RNAi plants to the bacterial leaf streak pathogen *Xoc*. The RNAi lines and wild‐type were inoculated with different *Xoc* strains at the tillering stage. The *Xoc* population was significantly lower in *OsImpα1a/1b*‐RNAi plants than in the wild‐type (Fig. 5C). The *OsImpα1a/1b*‐RNAi plants exhibited clearly shorter lesion length than the wild‐type after inoculation with different *Xoc* strains (Fig. 5D). Taken together, these results suggest that *OsImpα1a* and *OsImpα1b* negatively regulate rice resistance to the bacterial pathogens *Xoo* and *Xoc*.

### Activation of TALE‐targeted susceptibility genes is inhibited in *OsImpα1a*/*1b*‐RNAi plants

**Figure 6:**
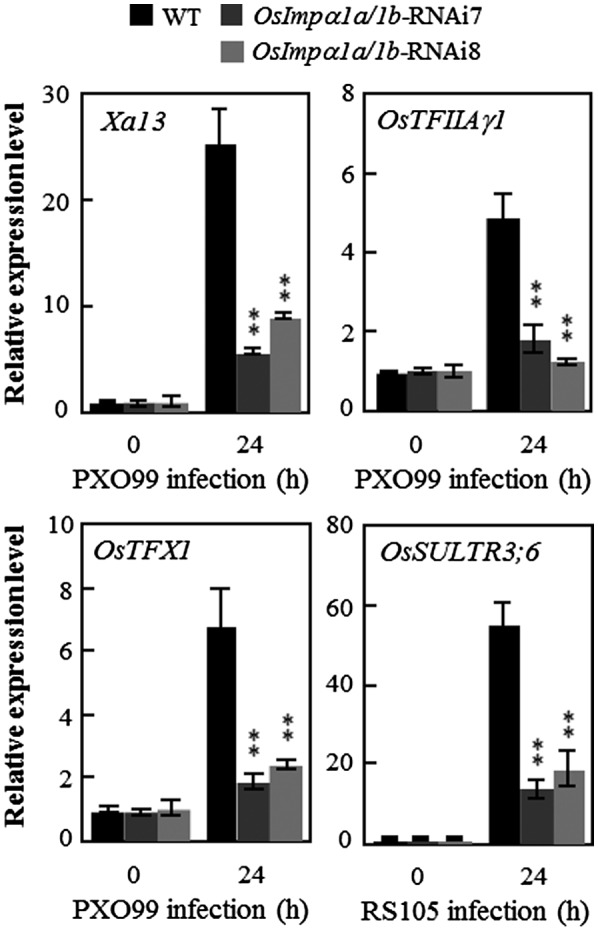
Effect of *OsImpα1a *and *OsImpα1b* on the expression of the disease susceptibility genes *Xa13*, *OsTFIIAγ1* and *OsTFX1* after *Xanthomonas oryzae* pv. *oryzae* (*Xoo*) infection, and *OsSULTR3;6* after *Xanthomonas oryzae* pv. *oryzicola *(*Xoc*) infection. Plants were inoculated with *Xoo* strain PXO99 [harbouring the transcription activator‐like effector (TALEs) pthXo1, pthXo6 and pthXo7] at the booting stage, or *Xoc* strain RS105 (harbouring TALE Tal2g) at the tillering stage. Asterisks indicate a significant difference between transgenic plants and wild‐type (WT) IR24 at **P < 0.01.

The bacterial pathogens *Xoo* and *Xoc* cause disease mainly through the targeting and activation of host rice susceptibility genes by their virulence TALEs. To monitor the expression pattern of rice TALE‐targeted susceptibility genes on *Xoo* and *Xoc* infection in plants, we inoculated *OsImpα1a/1b*‐RNAi plants with *Xoo* strain PXO99 at the booting stage and *Xoc* strain RS105 at the tillering stage. qRT‐PCR assays showed that the induced expression of the known susceptibility genes *Xa13*, *OsTFIIAγ1* and *OsTFX1*, each of which is targeted by a different TALE of *Xoo* PXO99, was significantly more suppressed in *OsImpα1a/1b*‐RNAi plants than in wild‐type plants (Fig. 6). Similarly, suppression of *OsImpα1a* and *OsImpα1b *in rice increased resistance to *Xoc*, which was associated with significantly hindered *Xoc*‐activated expression of the rice susceptibility gene *OsSULTR3;6*, which is targeted by a TALE Tal2g of *Xoc* RS105, compared with wild‐type plants (Fig. 6). These results demonstrate that the induction of TALE‐targeted susceptibility genes is largely inhibited in *OsImpα1a‐* and *OsImpα1b‐*suppressing plants after bacterial pathogen infection.

### Virulence of TALE‐free and T3SS‐free strains on *OsImpα1a*/*1b*‐RNAi plants

**Figure 7:**
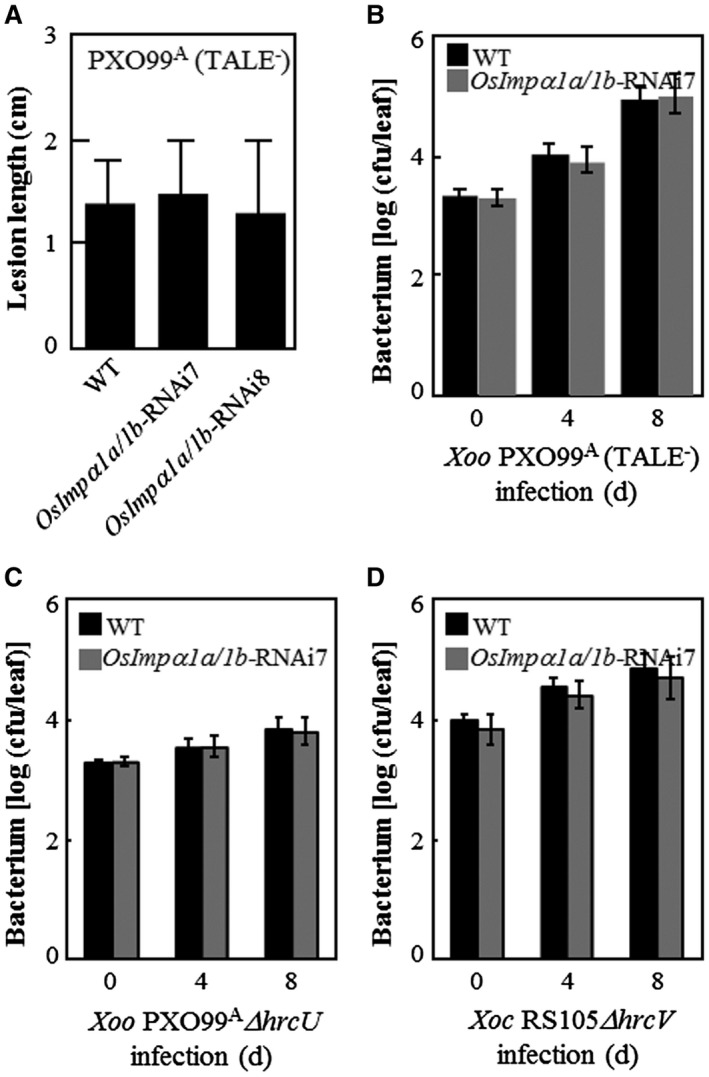
The virulence of transcription activator‐like effector (TALE)‐free and type III secretion system (T3SS)‐free bacterial pathogen strains on *OsImpα1a/1b*‐RNAi plants. Plants were inoculated with *Xanthomonas oryzae* pv. *oryzae* (*Xoo*) strain PXO99^A^ (TALE^–^) (TALE‐free strain, also named PH) and PXO99^A^ *ΔhrcU *(T3SS‐free strain) at the booting stage, or *Xanthomonas oryzae* pv. *oryzicola *(*Xoc*) strain RS105*ΔhrcV *(T3SS‐free strain) at the tillering stage. Data represent the mean (nine leaves from three plants) ± standard deviation (SD). (A) Lesion length of *OsImpα1a/1b*‐RNAi plants after inoculation with *Xoo* strain PXO99^A^ (TALE^–^). (B) Growth of *Xoo* strain PXO99^A^ (TALE^–^) in leaves of *OsImpα1a/1b*‐RNAi7 plants. cfu, colony‐forming unit. (C) Growth of *Xoo* strain PXO99^A^ *ΔhrcU *in leaves of *OsImpα1a/1b*‐RNAi7 plants. (D) Growth of *Xoc* strain RS105*ΔhrcV *in leaves of *OsImpα1a/1b*‐RNAi7 plants.

To assess whether the ability of *OsImpα1a* and *OsImpα1b* to confer resistance to *Xoo* and *Xoc* is exclusively associated with the virulence factors of bacterial pathogens, TALEs, but not non‐TALEs, we inoculated *OsImpα1a/1b*‐RNAi plants at the booting stage with *Xoo* strain PXO99^A^ (TALE^–^), also named PH (Ji *et al.*, 2016). The *OsImpα1a/1b*‐RNAi plants showed similar lesion lengths to the wild‐type (Fig. 7A), and the *Xoo* population was indistinguishable between leaves of *OsImpα1a/1b*‐RNAi plants and the wild‐type at 4 and 8 days after infection (Figs 7B, S7A, see Supporting Information). In addition, we inoculated *OsImpα1a/1b*‐RNAi plants and the wild‐type at the booting stage with *Xoo* strain PXO99^A^ *ΔhrcU*, which is a T3SS‐free strain lacking a functional T3SS and is unable to deliver TALEs and non‐TALEs into plant cells (Guo *et al.*, 2012). We found that there were similar *Xoo* populations in the leaves of *OsImpα1a/1b*‐RNAi plants and the wild‐type on the different days assessed after infection (Figs 7C, S7B). Furthermore, we simultaneously inoculated *OsImpα1a/1b*‐RNAi plants and the wild‐type at the tillering stage with *Xoc* strain RS105*ΔhrcV*, which is a T3SS‐free strain lacking a functional T3SS (Guo *et al.*, 2012). Similar *Xoc* populations were observed in the leaves of *OsImpα1a/1b*‐RNAi plants and the wild‐type on the different days evaluated after infection (Figs 7D, S7C). In brief, these data suggest that the broad‐spectrum resistance of *OsImpα1a/1b*‐RNAi plants to *Xoo* and *Xoc* relies on the existence of TALEs in bacterial pathogens.

### Mutational analysis of OsImpα1a/1b NLS binding sites

A previous study has validated that OsImpα1a has two separate NLS binding sites, the major site and the minor site, both of which recognize positively charged amino acid clusters in NLSs (Chang *et al.*, 2012). The residues D188 and E388 of OsImpα1a are essential for plant‐specific NLS binding to the major site and the minor site, respectively (Chang *et al.*, 2012). To investigate whether these two core residues of OsImpα1a are responsible for bacterial pathogen‐derived NLS binding, we produced OsImpα1a derivatives with the key residues D188 and E388 substituted by positively or negatively charged amino acid residues. These derivatives of OsImpα1a were co‐transformed into yeast with *Xoo*‐specific NLS to assess binding activity. Yeast two‐hybrid assays showed that mutation in the major site D188 with positively charged amino acid residues (D188E) or negatively charged amino acid residues (D188H, D188K, D188R), and mutation in the minor site E388 with positively charged amino acid residues (E388D) or negatively charged amino acid residues (E388H, E388K, E388R), did not attenuate the interaction between OsImpα1a and NLS2 of pthXo1 (Fig. S8A, see Supporting Information). In addition, the residues D194 and E394 of OsImpα1b, the key amino acids for NLS binding to the major site and the minor site, respectively, were assessed for interaction with *Xoo*‐derived NLS. Similarly, mutation in the major site D194 and in the minor site E394 did not influence OsImpα1b binding to *Xoo*‐specific NLS (Fig. S8B). In conclusion, the mutations in the major site and minor site of rice OsImpα1a and OsImpα1b do not have a noticeable impact on the binding affinity for *Xoo*‐ or *Xoc*‐derived NLS.

## Discussion

**Figure 8:**
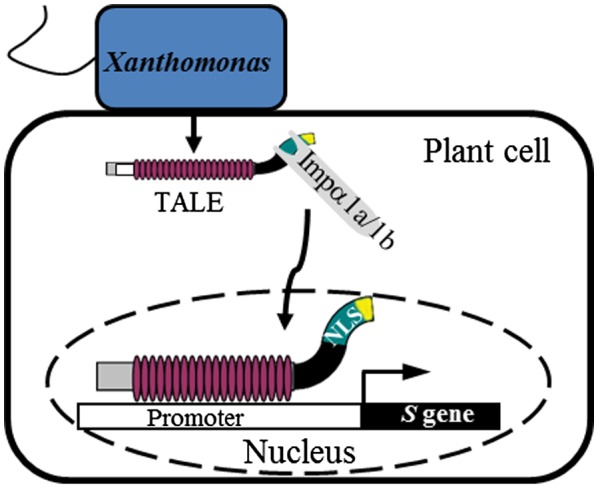
A model showing rice cytoplasm/nuclear shuttle protein Impa1a or Impa1b mediated transportation of TALEs from cytoplasm into nucleus is vital for TALE‐carrying bacterial pathogen triggered induction of targeting susceptibility (*S*) genes through direct interaction between plant Impa1a or Impa1b and nuclear localization signal 2 (NLS2) of TALEs. [Colour figure can be viewed at wileyonlinelibrary.com]

Previously, we have revealed that TALE‐carrying *Xoo* and *Xoc* hijack the host basal transcription factor IIA γ subunit (TFIIAγ) in the plant nucleus to cause disease in rice (Yuan *et al.*, 2016). Here, we further demonstrate that *Xoo* and *Xoc* capture host plant cytoplasm/nuclear shuttle proteins OsImpα1a and OsImpα1b for successful transfer of their virulence TALEs from the plant cytoplasm into the plant nucleus (Fig. 8). TALEs then interact with TFIIAγ, and bind and activate target susceptibility genes to cause disease in rice. This inference is supported by the following evidence. First, *Xoo* and *Xoc* employ their NLSs, specifically conserved NLS2, to interact with the host cytoplasm/nuclear shuttle proteins OsImpα1a and OsImpα1b. Second, transcriptional suppression of *OsImpα1a* and *OsImpα1b* enhances the resistance to diverse *Xoo* and *Xoc* strains, which is associated with the attenuated induction of susceptibility genes. Third, the efficient transportation of *Xoo* and *Xoc *TALEs into the plant nucleus is vital for bacterial pathogen invasion of rice.

The TALEs of isolated *Xoo* and *Xoc* strains with different geographical distributions all exclusively contain highly conserved NLS2, which is composed of five amino acid residues and is rich in arginine and lysine residues (RKRSR) (Booher *et al.*, 2015; Cernadas *et al.*, 2014). Although there are different amino acid residues of bacterial pathogen‐derived NLS2 and plant nuclear proteins contained T‐NLS and O_2_‐NLS, which two have been proved selectively binding by both OsImpα1a and OsImpα1b (Jiang *et al.*, 2001), TALEs of *Xoo* and *Xoc* mimic host nuclear proteins harbouring similar NLS for trapping by cytoplasm/nuclear shuttle proteins OsImpα1a and OsImpα1b, with the probably evidence that NLS2 of TALEs features with positively charged amino acids residues, lysine and arginine, which is accordance with T‐NLS and O_2_‐NLS. The exception to the transfer of plant‐derived proteins from the cytoplasm into the nucleus, OsImpα1a, can directly bind to NLSs of *Agrobacterium tumefaciens* virulence protein VirD2, which forms a protein–nucleic acid super‐complex with T‐DNA, facilitating T‐DNA nuclear import (Chang *et al.*, 2014). Moreover, the orthologues in pepper, CaImpα1 and CaImpα2, interact with TALE AvrBs3 of *Xcv* (Szurek *et al.*, 2001). Thus, bacterial pathogens *Xoo* and *Xoc*, like *Xcv*, have evolved the simplest NLSs to mimic plant‐derived nucleus‐localized proteins for the facilitation of the transportation of virulence TALEs into the plant nucleus for the infection of host rice.

In rice, at least three nucleus importin α proteins have been characterized with nucleus trafficking capacity. We found that OsImpα1a and OsImpα1b, but not OsImpα2, interact with NLS2 of TALEs in *in vitro* and *in vivo* assays. The possible mechanism is that these three cytoplasm/nuclear shuttle proteins are responsible for the transportation of different nucleus‐localized proteins by binding different types of NLS (Goldfarb *et al.*, 2004). In order to identify whether there is functional redundancy of OsImpα1a and OsImpα1b for TALE transportation, we used the clustered regularly interspersed short palindromic repeats (CRISPR)/Cas system to separately knock out these two genes, with both homozygotes of the *osImpα1a* and *osImpα1b* mutants being developmentally lethal. An RNAi strategy was used to suppress the transcript of these two genes, and *OsImpα1a/1b*‐RNAi plants showed slightly shorter flag leaf and panicle, fewer grains per panicle, decreased 1000‐grain weight and significantly less seed setting than wild‐type plants (Table S1, see Supporting Information). These data indicate that these two cytoplasm/nuclear shuttle proteins, OsImpα1a and OsImpα1b, probably play comparable and pivotal roles in the transportation of NLS‐containing proteins, which are essential for plant growth and development. Whether these two proteins play greater roles than their homologue OsImpα2 should be investigated further.

Rice importins α1a and α1b have been validated as components of the NLS receptor in plant cells, and transfer distinct groups of nuclear proteins (Jiang *et al.*, 1998, 2001). Here, we found that these two importin proteins also carry bacterial pathogen‐derived nuclear proteins into the plant nucleus. Our *in vitro* and *in vivo* assays showed that OsImpα1a and OsImpα1b associated with TALEs are dependent on the presence of NLS2, but not NLS1 or NLS3, which is in accordance with the analysis of TALE AvrBs3 of *Xcv*, where deletion of NLS1 or NLS3 of AvrBs3 is not sufficient to abolish the induction of the AvrBs3‐activated hypersensitive response (HR) on *Bs3*‐containing resistant pepper plants (Szurek *et al.*, 2001). NLS1, NLS2 and NLS3 of *Xanthomonas* are rich in positively charged amino acid residues. However, *Xanthomonas* species selectively use their NLS2, not NLS1 or NLS3, of TALEs to trap host plant cytoplasm/nuclear shuttle proteins. The functions of NLS1 and NLS3 during the process of *Xanthomonas* TALE‐triggered susceptibility in plants requires further study.

Both *OsImpα1a* and *OsImpα1b* were induced in rice leaves in response to infection with the bacterial pathogens *Xoo* or *Xoc*, whereas their homologous genes in pepper, *Caimpα1* and *Caimpα2*, were constitutively expressed independent of infection with *Xcv* (Szurek *et al.*, 2001). This diversity could be caused by differences between the respective host plants, rice and pepper. Plants with suppressed *OsImpα1a* and *OsImpα1b* transcription showed broad‐spectrum disease resistance to *Xoo* and *Xoc*, accompanied by attenuated induction of susceptibility genes. The reason that TALEs targeting susceptibility genes could not be immediately up‐regulated after *Xoo* and *Xoc* infection is a result of the inefficient transportation of TALEs into the plant nucleus. However, *OsImpα1a/1b*‐RNAi plants showed a similar lesion length and bacterial population to the wild‐type after inoculation with TALE‐free and T3SS‐free bacterial strains. These results further indicate that suppression of *OsImpα1a‐ *or *OsImpα1b*‐mediated resistance to *Xoo* or *Xoc* is solely associated with TALEs, the major virulence factors of bacterial pathogens.

In conclusion, the present results and a previous report (Szurek *et al.*, 2001) suggest that the TALE‐carrying bacterial pathogens, *Xoo*, *Xoc *and *Xcv*, use the same mechanism, i.e. the trapping of host plant nuclear import receptor proteins, to transfer their virulence TALEs from the plant cytoplasm into the nucleus to activate susceptibility genes to cause disease in rice and pepper. The TALE‐carrying genus *Xanthomonas* infects a wide range of plants (Jacques *et al.*, 2016), and the virulence TALEs contain an identical NLS2. The plants simultaneously carry nuclear import receptors with high amino acid sequence similarity (Wiermer *et al.*, 2007). Thus, moderate suppression of expression of plant nuclear import receptor proteins may provide an applicable strategy to improve disease resistance to TALE‐carrying bacterial pathogens in other plants.

## Experimental Procedures

### Plant and bacterial materials

Rice (*Oryza sativa* ssp. *Xian*) IR24 is susceptible to *Xoo* and *Xoc*, and was used in this study. Plants were grown during a normal rice‐growing season under natural field conditions.

The reference *Xoo* strain PXO99^A^ and its mutation PH [PXO99^A^ (TALE^–^)], with deletion of all 19 TALE genes, and *Xoc* strain RS105 have commonly been used in studies of rice resistance to bacterial leaf blight disease and bacterial leaf streak disease, respectively (Ji *et al.*, 2016), and were used in this study. *Xoo* strain PXO99 was used for pathogen inoculation. PXO99^A^, a 5‐azacytidine‐resistant mutant of PXO99, was used for genetic manipulation and pathogen inoculation. All the *Xanthomonas* strains were grown at 28 °C on nutrient agar medium. When genetic manipulation of bacteria was undertaken, antibiotics were used at the following final concentrations as required: ampicillin, 100 μg/mL; rifampicin, 75 μg/mL; kanamycin, 25 μg/mL.

### Site‐directed mutation

Site mutation of plant *OsImpα1a* and *OsImpα1b* genes and *Xoo* *pthXo1* gene was performed using the GeneTailor Site‐Directed Mutagenesis System (Invitrogen Life Technologies, Carlsbad, CA, USA), as described previously (Yuan *et al.*, 2009). The site‐mutated genes were confirmed by Sanger sequencing.

### Vector construction and plant transformation

The TALE pthXo1 and its variations were cloned into the pHM1 vector to produce pHM1‐pthXo1, and then transferred into *Xoo* strain PH following a published method (Ji *et al.*, 2016). The NLS2 region of pthXo1 was replaced with its site‐directed mutations by Gibson assembly (Gibson *et al.*, 2009), following confirmation by Sanger sequencing.

The full‐length cDNAs of *OsImpα1a*, *OsImpα1b* and *OsNMD3* were ligated into the pU1301‐3FLAG vector. The recombinant vectors were introduced into *A. tumefaciens* strain GV3101. *Agrobacterium*‐mediated transformation was performed by infiltration into *N. benthamiana* leaves using a needleless syringe (Yuan *et al.*, 2016).

To construct the RNAi vector, the gene‐specific and high‐similarity fragment of *OsImpα1a* and *OsImpα1b* genes was amplified and inserted into the pDS1301 vector (Yuan *et al.*, 2010). The recombinant vector was introduced into *A. tumefaciens* strain EHA105. *Agrobacterium*‐mediated transformation was performed using calli derived from mature embryos of rice variety IR24, according to a published protocol (Ge *et al.*, 2006).

### Protein–protein interaction

To study the interaction between *Xanthomonas* TALEs and rice OsImpα1a, OsImpα1b and OsImpα2 in yeast two‐hybrid assays, the different domains of TALE pthXo1 and its variations were amplified using gene‐specific primers (Yuan *et al.*, 2016); the amplified DNA segments were then ligated into the pGBKT7 vector. Rice *OsImpα1a*, *OsImpα1b*, *OsImpα2* and their variations were amplified using gene‐specific primers (Table S2, see Supporting Information), and the amplified DNA segments were ligated into the pGADT7‐Rec vector. The recombinant pGBKT7 and pGADT7 plasmids were then co‐transformed into yeast strain AH109 for interaction analyses (Yuan *et al.*, 2010). The yeast clones were restreak on synthetic defined premixed (SD) medium lacking leucine (L) and tryptophan (W) (−LW) and selective SD medium lacking L, W, histidine (H) and adenine (A) (−LWHA).

To study the interaction *in planta*, Co‐IP assays were performed (Yuan *et al.*, 2016). The DNA segments of pthXo1 and its variations were ligated into the pU1301‐9myc vector; the DNA segments of *OsImpα1a*, *OsImpα1b* and *OsImpα2* were ligated into the pU1301‐9myc vector (Yuan *et al.*, 2010). The recombinant constructs were introduced into *A. tumefaciens* strain GV3101 by electroporation. *Agrobacterium*‐mediated transformation was performed by infiltration into *N. benthamiana* leaves using a needleless syringe. Co‐IP assays were carried out using anti‐FLAG antibody (F7425, Sigma, Sigma‐Aldrich, St. Louis, Missouri, USA) and anti‐myc antibody (AB103, Tiangen, Beijing, China), as described previously (Yuan *et al.*, 2016). Each Co‐IP assay was repeated at least twice.

### Split‐luciferase complementation assay

To construct split‐luciferase complementation assay vector, the open reading frame of *pthXo1* was inserted into the vector pCAMBIA‐35S‐nLUC to generate construct pthXo1‐nLUC; the open reading frames of *OsImpα1a*, *OsImpα1b *and *OsImpα2 *were inserted into the vector pCAMBIA‐35S‐cLUC to generate constructs OsImpα1a‐cLUC, OsImpα1b‐cLUC and OsImpα2‐cLUC, respectively. The recombinant constructs were introduced into *A. tumefaciens* strain GV3101. Equal amounts of *Agrobacterium *cultures for nLUC and cLUC constructs were mixed and co‐transformed into fully expanded leaves of *N. benthamiana*. After 2 days, 1 mm of precooled luciferin was sprayed onto the leaves, and the samples were incubated in the dark for 5 min. LUC images were captured using a cooled charge coupled device (CCD) imaging apparatus (Chen *et al.*, 2008).

### Pathogen inoculation

To evaluate rice bacterial blight disease, five to seven uppermost fully expanded leaves of each plant were inoculated with different *Xoo* strains at an optical density at 600 nm (OD_600nm_) = 0.5 by the leaf clipping method (Yuan *et al.*, 2016) at the booting (panicle development) stage. *Xoo* strains included Philippine strains PXO61, PXO86, PXO71, PXO112, PXO99, PXO347 and PXO341, Chinese strains Zhe173, KS‐1‐21, YN11 and FuJ23, Japanese strain T7174 and Korean strain KACC10331. Disease was scored by measuring the lesion length at 14 days after inoculation. The bacterial growth rate in rice leaves was determined by counting the number of colony‐forming units (Yuan *et al.*, 2016).

To evaluate rice bacterial streak disease, five to eight fully expanded leaves were inoculated with *Xoc* strains with OD_600nm_ = 0.5 by the penetration method at the tillering stage (Yuan *et al.*, 2016). *Xoc* strains included Chinese strains RH3, RS85, RS105, JSB2‐24 and HNB8‐47. The disease was scored by measuring the lesion length at 14 days after inoculation. The bacterial growth rate in rice leaves was determined by counting the number of colony‐forming units (Yuan *et al.*, 2016).

For the measurement of the growth *in planta* of *Xoo* strains PXO99^A^ (TALE^–^) and PXO99^A^ *ΔhrcU*, and *Xoc* strain RS105*ΔhrcV*, these pathogens were infiltrated into the intercellular spaces of fully expanded rice leaves with needleless syringes at three different locations per leaf. The bacterial growth rate in rice leaves was determined by counting the number of colony‐forming units (Yuan *et al.*, 2016).

### Gene expression analysis

For gene expression analysis, real‐time qRT‐PCR was performed using SYBR Premix Ex Taq (Takara, Dalian, China) in an ABI 7500 Real‐Time PCR System (Applied Biosystems, Foster, California, USA). In brief, 2‐cm rice leaf fragments near the bacterial infection sites were collected for RNA isolation. Total RNA was extracted using Trizol reagent (Invitrogen, Carlsbad, California, USA). An aliquot (5 μg) of total RNA was treated with RNase‐free DNase I (Invitrogen) to remove potentially contaminating DNA, and first‐strand cDNA was reverse transcribed from total RNA with oligo(dT)_18_ primer using M‐MLV reverse transcriptase (Promega, Madison, Wisconsin, USA) according to the manufacturer’s instructions. qRT‐PCR was conducted using gene‐specific primers (Table S3, see Supporting Information). The expression level of the rice *actin* gene was used to standardize the RNA sample as an internal control. The expression level relative to that of controls was assessed. Each qRT‐PCR assay was repeated at least twice with a similar result, with each repetition having three replicates.

### Western blotting

The nuclear proteins were extracted as described previously (Moes *et al.*, 2013). The protein samples were separated on a sodium dodecylsulfate‐polyacrylamide gel electrophoresis (SDS‐PAGE) gel, transferred onto a nitrocellulose membrane and then analysed by blotting with different antibodies. The antibodies used for immunoblotting analyses included anti‐FLAG (F7425, Sigma), anti‐Histone H3 (06‐755, Millipore, Burlington, Massachusetts) and anti‐PEPC (AS09458, Agrisera, Vannas, Sweden).

### Sequence analysis

Multiple sequence alignments of amino acid sequences were generated using ClustalW in MEGA X with the default parameters. The sequence alignments obtained were used as input for the neighbour‐joining analysis in MEGA X to construct the phylogenetic tree. For phylogenetic tree construction, a bootstrap method with 1000 replications was used for test of phylogeny, with Poisson model and pairwise deletion during gaps/missing date treatment.

### Statistical analysis

Differences between samples were analysed for statistical significance using SPSS software and Student’s *t*‐test (two‐tailed). The correlation analysis between disease symptom and gene expression level was analysed using the Pearson correlation coefficient analysis in SPSS (IBM SPSS Statistics, Version 19.0, IBM Corp., released 2010, Armonk, New York).

## Supporting information


**Fig. S1 **Phylogenetic tree of plant importin α proteins. Sequences were analysed by the neighbour‐joining method with genetic distance calculated by MEGA X. OsImpα1a (XP_015621115), OsImpα1b (XP_015639761) and OsImpα2 (XP_015619230) from *Oryza sativa*; CaImpα1 (AAK38726) and CaImpα2 (AAK38727) from *Capsicum annuum*; LeKAPα1 (AAC23722) from *Solanum lycopersicum*; AtImpα1 (NP_187328), AtImpα2 (NP_001154239), AtImpα3 (NP_192124) and AtImpα4 (NP_172398) from *Arabidopsis thaliana*; CsImpα1a (XP_006480039), CsImpα1b (XP_006472169) and CsImpα2 (XP_006488879) from *Citrus sinensis*; GhImpα1 (XP_016667887) and GhImpα2 (XP_016695962) from *Gossypium hirsutum*; PvImpα (XP_007142798) from *Phaseolus vulgaris*.

**Fig. S2 **NLS2 of pthXo1 interacts with rice OsImpα1a and OsImpα1b in yeast cells by yeast two‐hybrid assay. The interactions were assessed by the growth of yeast cells on synthetic defined premixed (SD) medium lacking (–) leucine (L), tryptophan (W), histidine (H) and adenine (A). Vector, empty vector as control; RR, repeat region; TFB, transcription factor binding region; NLS, nuclear localization signal. (A) NLS2 of pthXo1 interacts with OsImpα1a. (B) NLS2 of pthXo1 interacts with OsImpα1b. (C) NLS2 of pthXo1 does not interact with OsImpα2.

**Fig. S3 **Interaction between point‐mutated NLS2 of pthXo1 and OsImpα1b analysed by yeast two‐hybrid assay. The interactions were assessed by the growth of yeast cells on synthetic defined premixed (SD) medium lacking (–) leucine (L), tryptophan (W), histidine (H) and adenine (A).

**Fig. S4 **Expression of *OsImpα1a* and *OsImpα1b* after infection with *Xanthomonas oryzae* pv. *oryzicola (Xoc)* strain RH3 at the tillering stage.

**Fig. S5 **Nucleotide sequence alignment of *OsImpα1a* and *OsImpα1b* by MUSCLE.

**Fig. S6** Analysis of the response of two *OsImpα1a/1b*‐RNAi T1 families to *Xanthomonas oryzae* pv. *oryzae (Xoo)* strain PXO99. Data represent the mean (five to eight leaves from one plant for lesion length) ± standard deviation (SD). Asterisks indicate a significant difference between transgenic plants and wild‐type (WT) IR24 at **P < 0.01.

**Fig. S7 **The virulence of transcription activator‐like effector (TALE)‐free and type III secretion system (T3SS)‐free bacterial pathogen strains on *OsImpα1a/1b*‐RNAi8 plants. Plants were inoculated with *Xanthomonas oryzae* pv. *oryzae (Xoo)* strain PXO99^A^ (TALE^-^) (TALE‐free strain, also named PH) and PXO99^A^ *hrcU* (T3SS‐free strain) at the booting stage, or *Xanthomonas oryzae* pv. *oryzicola (Xoc)* strain RS105*Δ* *hrcV* (T3SS‐free strain) at the tillering stage. Data represent the mean (nine leaves from three plants) ± standard deviation (SD). (A) Growth of *Xoo* strain PXO99^A^ (TALE^-^) in leaves of *OsImpα1a/1b*‐RNAi8 plants. (B) Growth of *Xoo* strain PXO99^A^ *Δ* *DhrcU* in leaves of *OsImpα1a/1b*‐RNAi8 plants. (C) Growth of *Xoc *strain RS105*Δ* *hrcV *in leaves of *OsImpα1a/1b*‐RNAi8 plants.

**Fig. S8 **Interaction between NLS2 of pthXo1 and point‐mutated OsImpα1a (A) or OsImpα1b (B) analysed by yeast two‐hybrid assay. The interactions were assessed by the growth of yeast cells on synthetic defined premixed (SD) medium lacking (–) leucine (L), tryptophan (W), histidine (H) and adenine (A).

**Table S1 **Measurements of agronomic traits of *OsImpα1a/1b*‐RNAi plants under natural field conditions

**Table S2 **Polymerase chain reaction (PCR) primers used for the construction of vectors for transformation and protein–protein interactions.

**Table S3 **Polymerase chain reaction (PCR) primers used for quantitative reverse transcription (RT)‐PCR assays.
