# Supplementary material for: TALE‐carrying bacterial pathogens trap host nuclear import receptors for facilitation of infection of rice
Source: Mol Plant Pathol. 2019 Jan 9;20(4):519–32. doi: 10.1111/mpp.12772 (PMC6637887; doi:10.1111/mpp.12772)
Supplement: Supplementary file 1 — Fig. S1 Phylogenetic tree of plant importin α proteins. Sequences were analysed by the neighbour‐joining method with genetic distance calculated by MEGA X. OsImpα1a (XP_015621115), OsImpα1b (XP_015639761) and OsImpα2 (XP_015619230) from Oryza sativa; CaImpα1 (AAK38726) and CaImpα2 (AAK38727) from Capsicum annuum; LeKAPα1 (AAC23722) from Solanum lycopersicum; AtImpα1 (NP_187328), AtImpα2 (NP_001154239), AtImpα3 (NP_192124) and AtImpα4 (NP_172398) from Arabidopsis thaliana; CsImpα1a (XP_006480039), CsImpα1b (XP_006472169) and CsImpα2 (XP_006488879) from Citrus sinensis; GhImpα1 (XP_016667887) and GhImpα2 (XP_016695962) from Gossypium hirsutum; PvImpα (XP_007142798) from Phaseolus vulgaris. [file MPP-20-519-s001.docx]

**Fig. S1** Phylogenetic tree of plant importin α proteins.

Sequences were analyzed by the neighbor-joining method with genetic distance calculated by MEGA X. OsImpα1a (XP_015621115), OsImpα1b (XP_015639761) and OsImpα2 (XP_015619230) from *Oryza sativa*; CaImpα1 (AAK38726) and CaImpα2 (AAK38727) from *Capsicum annuum*; LeKAPα1 (AAC23722) from *Solanum lycopersicum*; AtImpα1 (NP_187328), AtImpα2 (NP_001154239), AtImpα3 (NP_192124) and AtImpα4 (NP_172398) from *Arabidopsis thaliana*; CsImpα1a (XP_006480039), CsImpα1b (XP_006472169) and CsImpα2 (XP_006488879) from *Citrus sinensis*; GhImpα1 (XP_016667887) and GhImpα2 (XP_016695962) from *Gossypium hirsutum*; PvImpα (XP_007142798) from *Phaseolus vulgaris*.
