# Supplementary material for: TALE‐carrying bacterial pathogens trap host nuclear import receptors for facilitation of infection of rice
Source: Mol Plant Pathol. 2019 Jan 9;20(4):519–32. doi: 10.1111/mpp.12772 (PMC6637887; doi:10.1111/mpp.12772)
Supplement: Supplementary file 2 — Fig. S2 NLS2 of pthXo1 interacts with rice OsImpα1a and OsImpα1b in yeast cells by yeast two‐hybrid assay. The interactions were assessed by the growth of yeast cells on synthetic defined premixed (SD) medium lacking (–) leucine (L), tryptophan (W), histidine (H) and adenine (A). Vector, empty vector as control; RR, repeat region; TFB, transcription factor binding region; NLS, nuclear localization signal. (A) NLS2 of pthXo1 interacts with OsImpα1a. (B) NLS2 of pthXo1 interacts with OsImpα1b. (C) NLS2 of pthXo1 does not interact with OsImpα2. [file MPP-20-519-s002.docx]

**Fig. S2** NLS2 of pthXo1 interacts with rice OsImpα1a and OsImpα1b in yeast cells by yeast two-hybrid assay. The interactions were assessed by growth of yeast cells on synthetic defined premixes (SD) medium lacking (-) leucine (L), tryptophan (W), histidine (H), and adenine (A). Vector, empty vector as control. RR, repeat region; TFB, transcription factor binding region; NLS, nuclear localization signal. (A) NLS2 of pthXo1 interacts with OsImpα1a. (B) NLS2 of pthXo1 interacts with OsImpα1b. (C) NLS2 of pthXo1 does not interact with OsImpα2.
