# Supplementary material for: TALE‐carrying bacterial pathogens trap host nuclear import receptors for facilitation of infection of rice
Source: Mol Plant Pathol. 2019 Jan 9;20(4):519–32. doi: 10.1111/mpp.12772 (PMC6637887; doi:10.1111/mpp.12772)
Supplement: Supplementary file 4 — Fig. S4 Expression of OsImpα1a and OsImpα1b after infection with Xanthomonas oryzae pv. oryzicola (Xoc) strain RH3 at the tillering stage. [file MPP-20-519-s004.docx]

**Fig. S4** Expression of *OsImpα1a* and *OsImpα1b* after infection with *Xoc* strain RH3 at the tillering stage.
