# Supplementary material for: TALE‐carrying bacterial pathogens trap host nuclear import receptors for facilitation of infection of rice
Source: Mol Plant Pathol. 2019 Jan 9;20(4):519–32. doi: 10.1111/mpp.12772 (PMC6637887; doi:10.1111/mpp.12772)
Supplement: Supplementary file 5 — Fig. S5 Nucleotide sequence alignment of OsImpα1a and OsImpα1b by MUSCLE. [file MPP-20-519-s005.docx]

**Fig. S5** Nucleotide sequence alignment of *OsImpα1a* and *OsImpα1b* by MUSCLE.
