# Supplementary material for: TALE‐carrying bacterial pathogens trap host nuclear import receptors for facilitation of infection of rice
Source: Mol Plant Pathol. 2019 Jan 9;20(4):519–32. doi: 10.1111/mpp.12772 (PMC6637887; doi:10.1111/mpp.12772)
Supplement: Supplementary file 6 — Fig. S6 Analysis of the response of two OsImpα1a/1b‐RNAi T1 families to Xanthomonas oryzae pv. oryzae (Xoo) strain PXO99. Data represent the mean (five to eight leaves from one plant for lesion length) ± standard deviation (SD). Asterisks indicate a significant difference between transgenic plants and wild‐type (WT) IR24 at **P < 0.01. [file MPP-20-519-s006.docx]

**Fig. S6** Analysis of two *OsImpα1a/1b*-RNAi T_1_ families to *Xoo* strain PXO99. Data represent mean (five to eight leaves from one plant for lesion length) ± SD. Asterisks indicate a significant difference between transgenic plants and wild-type (WT) IR24 at ***P* < 0.01.
