# Supplementary material for: TALE‐carrying bacterial pathogens trap host nuclear import receptors for facilitation of infection of rice
Source: Mol Plant Pathol. 2019 Jan 9;20(4):519–32. doi: 10.1111/mpp.12772 (PMC6637887; doi:10.1111/mpp.12772)
Supplement: Supplementary file 7 — Fig. S7 The virulence of transcription activator‐like effector (TALE)‐free and type III secretion system (T3SS)‐free bacterial pathogen strains on OsImpα1a/1b‐RNAi8 plants. Plants were inoculated with Xanthomonas oryzae pv. oryzae (Xoo) strain PXO99A (TALE-) (TALE‐free strain, also named PH) and PXO99A hrcU (T3SS‐free strain) at the booting stage, or Xanthomonas oryzae pv. oryzicola (Xoc) strain RS105Δ hrcV (T3SS‐free strain) at the tillering stage. Data represent the mean (nine leaves from three plants) ± standard deviation (SD). (A) Growth of Xoo strain PXO99A (TALE-) in leaves of OsImpα1a/1b‐RNAi8 plants. (B) Growth of Xoo strain PXO99A Δ DhrcU in leaves of OsImpα1a/1b‐RNAi8 plants. (C) Growth of Xoc strain RS105Δ hrcV in leaves of OsImpα1a/1b‐RNAi8 plants. [file MPP-20-519-s007.docx]

**Fig. S7** The virulence of TALE-free and T3SS-free bacterial pathogen strains on *OsImpα1a/1b*-RNAi8 plants. Plants were inoculated with *Xoo* strain PXO99^A^ (TALE^-^) (TALE-free strain, also named PH) and PXO99^A^*ΔhrcU* (T3SS-free strain) at the booting stage, or *Xoc* strain RS105*ΔhrcV* (T3SS-free strain) at the tillering stage. Data represent mean (nine leaves from three plants) ± SD. (A) Growth of *Xoo* strain PXO99^A^ (TALE^-^) in leaves of *OsImpα1a/1b*-RNAi8 plants. (B) Growth of *Xoo* strain PXO99^A^*ΔhrcU* in leaves of *OsImpα1a/1b*-RNAi8 plants. (C) Growth of *Xoc* strain RS105*ΔhrcV* in leaves of *OsImpα1a/1b*-RNAi8 plants.
