# Supplementary material for: TALE‐carrying bacterial pathogens trap host nuclear import receptors for facilitation of infection of rice
Source: Mol Plant Pathol. 2019 Jan 9;20(4):519–32. doi: 10.1111/mpp.12772 (PMC6637887; doi:10.1111/mpp.12772)
Supplement: Supplementary file 8 — Fig. S8 Interaction between NLS2 of pthXo1 and point‐mutated OsImpα1a (A) or OsImpα1b (B) analysed by yeast two‐hybrid assay. The interactions were assessed by the growth of yeast cells on synthetic defined premixed (SD) medium lacking (–) leucine (L), tryptophan (W), histidine (H) and adenine (A). [file MPP-20-519-s008.docx]

**Fig. S8** Interaction between NLS2 of pthXo1 and point mutated OsImpα1a (A) or OsImpα1b (B) analyzed in yeast by yeast two hybrid assay. The interactions were assessed by growth of yeast cells on synthetic defined premixes (SD) medium lacking (-) leucine (L), tryptophan (W), histidine (H), and adenine (A).
