# Supplementary material for: TALE‐carrying bacterial pathogens trap host nuclear import receptors for facilitation of infection of rice
Source: Mol Plant Pathol. 2019 Jan 9;20(4):519–32. doi: 10.1111/mpp.12772 (PMC6637887; doi:10.1111/mpp.12772)
Supplement: Supplementary file 9 — Table S1 Measurements of agronomic traits of OsImpα1a/1b‐RNAi plants under natural field conditions [file MPP-20-519-s009.docx]

**Table S1.** Measurements of agronomic traits of *OsImpα1a/1b*-RNAi plants under natural field conditions

| Phenotype | IR24 | *OsImpα1a/1b*-RNAi7 | *OsImpα1a/1b*-RNAi8 |
| --- | --- | --- | --- |
| Heading date (days) | 95.00 ± 3.00 | 92.50 ± 4.50 | 93.00 ± 2.50 |
| Plant height (cm) | 91.22 ± 2.31 | 89.42 ± 3.08 | 90.41 ± 3.44 |
| Flag leaf length (cm) | 37.60 ± 1.66 | 33.65 ± 2.55* | 31.54 ± 1.84** |
| Number of panicles per plant | 15.00 ± 1.90 | 14.20 ± 2.10 | 14.60 ± 1.60 |
| Panicle length (cm) | 22.32 ± 0.53 | 16.56 ± 0.32** | 14.89 ± 0.93** |
| Grains per panicle | 175.50 ± 9.60 | 124.50 ± 6.70** | 114.80 ± 9.06** |
| 1000-grain weight (g) | 23.86 ± 2.18 | 16.72 ± 1.84** | 15.93 ± 2.09** |
| Seed setting (%) | 92.70 ± 5.30 | 75.60 ± 3.80** | 72.96 ± 7.80** |

Asterisks indicate a significant difference between transgenic plants and wild type (WT) IR24 at **P* < 0.05 or ***P* < 0.01.
