# Supplementary material for: TALE‐carrying bacterial pathogens trap host nuclear import receptors for facilitation of infection of rice
Source: Mol Plant Pathol. 2019 Jan 9;20(4):519–32. doi: 10.1111/mpp.12772 (PMC6637887; doi:10.1111/mpp.12772)
Supplement: Supplementary file 10 — Table S2 Polymerase chain reaction (PCR) primers used for the construction of vectors for transformation and protein–protein interactions. [file MPP-20-519-s010.docx]

**Table S2.** PCR primers used for construction of vectors for transformation and protein–protein interactions.

| Gene (accession number) | Forward primer (5’-3’) | Reverse primer (5’-3’) | Use |
| --- | --- | --- | --- |
| *OsImpa1a*  (AK068233) | AGACTAGT^a^GGTACC^b^AGAAAGAGGCTTGC | AAGAGCTC^c^GGATCC^d^GAATTGAGCAGCACCACC | Amplifying cDNA fragment for constructing RNAi construct |
|  | CCGGAATTC^e^ATGTCGCTGCGCCCGAGCGAG | CGCGGATCC^d^TTATTTGAATTGAGCAGCACC | Amplifying cDNA fragment for constructing yeast two hybrid construct |
|  | CGGGGTACC^b^ATGTCGCTGCGCCCGAGCGAG | CGCGGATCC^d^TTATTTGAATTGAGCAGCACC | Amplifying cDNA fragment for constructing split luciferase complementation construct |
|  | CGGGGTACC^b^ATGTCGCTGCGCCCGAGCGAG | CGCGGATCC^d^TTATTTGAATTGAGCAGCACC | Amplifying cDNA fragment for constructing expressing construct |
| *OsImpa1b*  (AK100133) | CCGGAATTC^e^ATGTCGCTGCGGCCGAGC | CGCGGATCC^d^TCAGCCAAAGTTGAATCC | Amplifying cDNA fragment for constructing yeast two hybrid construct |
|  | CGGGGTACC^b^ATGTCGCTGCGGCCGAGC | CGCGGATCC^d^TCAGCCAAAGTTGAATCC | Amplifying cDNA fragment for constructing split luciferase complementation construct |
|  | CGGGGTACC^b^ATGTCGCTGCGGCCGAGC | CGCGGATCC^d^TCAGCCAAAGTTGAATCC | Amplifying cDNA fragment for constructing expressing construct |
| *OsImpa2*  (AK103127) | CCGGAATTC^e^ATGGCCGACGACAGCGCC | CGCGGATCC^d^TCACTCGTCGAGGCCATAATCC | Amplifying cDNA fragment for constructing yeast two hybrid construct |
|  | CGGGGTACC^b^ATGGCCGACGACAGCGCC | CGCGGATCC^d^TCACTCGTCGAGGCCATAATCC | Amplifying cDNA fragment for constructing split luciferase complementation construct |
|  | CGGGGTACC^b^ATGGCCGACGACAGCGCC | CGCGGATCC^d^TCACTCGTCGAGGCCATAATCC | Amplifying cDNA fragment for constructing expressing construct |
| *OsNMD3*  (AK060642) | CGGGGTACC^b^ATGCTGCCGGGGTCGGCG | CGCGGATCC^d^TCATCCCACCATAACAGCATG | Amplifying cDNA fragment for constructing expressing construct |
| *pthXo1*  (CP000967) | TCCCCCGGG^f^GTGCAATCGGGTCTGC | TCCCCCGGG^f^TCAGATCGTCCCTCCGAC | Amplifying cDNA fragment for constructing split luciferase complementation construct |

^a^The underlined nucleotides are the digestion site of *Spe*I.

^b^The underlined nucleotides are the digestion site of *Kpn*I.

^c^The underlined nucleotides are the digestion site of *Sac*I.

^d^The underlined nucleotides are the digestion site of *Bam*HI.

^e^The underlined nucleotides are the digestion site of *Eco*RI.

^f^The underlined nucleotides are the digestion site of *Sma*I.
