# Supplementary material for: TALE‐carrying bacterial pathogens trap host nuclear import receptors for facilitation of infection of rice
Source: Mol Plant Pathol. 2019 Jan 9;20(4):519–32. doi: 10.1111/mpp.12772 (PMC6637887; doi:10.1111/mpp.12772)
Supplement: Supplementary file 11 — Table S3 Polymerase chain reaction (PCR) primers used for quantitative reverse transcription (RT)‐PCR assays. [file MPP-20-519-s011.docx]

**Table S3.** PCR primers used for quantitative RT-PCR assays.

| Gene (GenBank accession no.) | Forward primer (5’-3’) | Reverse primer (5’-3’) |
| --- | --- | --- |
| *Xa13*  (DQ421395) | TGGTTCTGCTACGGCCTCTT | GGTACCAGAAGTAGAGCCCCATCT |
| *OsImpa1a* (AK068233) | CGTGATTGAAGCTGGTGTTTG | CAGTTCGTAGTGCGGGTATAAG |
| *OsImpα1b* (AK100133) | GTAATGTCAGTCCCAGTCCTTG | GAGACAGAGCCCAAGTAAATCC |
| *OsTFIIAγ1* (CB097192) | CGAGCTCGCCATCCAAGT | TGTGCAGATGGCCCTTGAC |
| *OsTFX1* (AK108319) | CCCACTACCACAGCAACATGA | CACAGGTAGCTGCTGGGAAGT |
| *OsSULTR3;6* (AK121195) | TGGCGATGGTCAAGAACGA | TGATACCAAACGCGATCATCTC |
| *Osactin*  (X15865) | TGTATGCCAGTGGTCGTACCA | CCAGCAAGGTCGAGACGAA |
